# Long-term adherence and effects on grip strength and upper leg performance of prescribed supplemental vitamin D in pregnant and recently pregnant women of Somali and Swedish birth with 25-hydroxyvitamin D deficiency: a before-and-after treatment study

**DOI:** 10.1186/s12884-016-1117-3

**Published:** 2016-11-15

**Authors:** Paul Kalliokoski, Nils Rodhe, Yngve Bergqvist, Monica Löfvander

**Affiliations:** 1 Primary Health Care Centre Jakobsgårdarna, Jaxtorget 7A, Box 100 33, S-781 10 Borlänge, Sweden; 2 Centre for Clinical Research Dalarna, Falun, Sweden; 3 Uppsala University and Centre for Clinical Research Västmanland, Västerås, Sweden; 4 Division of Family Medicine, Department of Neurobiology, Care Sciences and Society, Karolinska Institutet, Huddinge, Sweden; 5 Department of Public Health and Caring Sciences, Family Medicine and Preventive Medicine Section, Uppsala University, Uppsala, Sweden

**Keywords:** Adherence, Multicultural care, Vitamin D, Physical performance, Grip strength, Pregnancy, Somalia

## Abstract

**Background:**

Muscular weakness and severe vitamin D deficiency is prevalent in Somali (veiled) pregnant women, Sweden. The study aims here were to explore adherence to prescribed supplemental vitamin D in new mothers with vitamin D deficiency and its effects on grip strength and upper leg performance in Somali (target group TG) and Swedish women (reference group RG) from spring through winter.

**Methods:**

A before- and after study was designed. A cross-sectional sample of women in antenatal care with serum 25-OHD ≤50 nmol/L were prescribed one or two tablets daily (800 or 1600 IU vitamin D3 with calcium) for 10 months. Reminders were made by Somali nurses (TG) or Swedish doctors (RG). Baseline and 10 month measurements of plasma nmol/L 25-OHD, maximal grip strength held for 10 s (Newton, N) and ability to squat (yes;no) were done. Total tablet intake (n) was calculated. Outcome variables were changes from baseline in grip strength and ability to squat. Predicting variables for change in grip strength and ability to squat were calculated using linear and binary regression in final models. Undetectable 25-OHD values (<10 nmol/L) were replaced with ‘9’ in statistic calculations.

**Results:**

Seventy-one women (46 TG, 1/3 with undetectable baseline 25-OHD; 25 RG) participated. At the 10-month follow up, 17% TG and 8% RG women reported having refrained from supplement. Mean 25-OHD increased 16 to 49 nmol/L (TG) and 39 nmol/L to 67 nmol/L (RG), (both *p* < 0.001). Grip strength had improved from 153 to 188 N (TG) (*p* < 0.001) and from 257 to 297 N (RG) (*p* = 0.003) and inability to squat had decreased in TG (35 to 9, *p* < 0.001). Intake of number of tablets predicted increased grip strength (B 0.067, 95%CI 0.008–0.127, *p* = 0.027). One tablet daily (>300 in total) predicted improved ability to squat (OR 16; 95% CI 1.8–144.6).

**Conclusions:**

Adherence to supplemental vitamin D and calcium should be encouraged as an even moderate intake was associated to improved grip strength and upper leg performance, which was particularly useful for the women with severe 25-OHD deficiency and poor physical performance at baseline.

**Trial registration:**

ClinicalTrials.gov Identifier: NCT02922803. Date of registration: September 28, 2016.

## Background

Muscular strength is crucial for delivering and caring an infant. However, some women have weak muscles and what do we do for them? An increased muscle power, often measured as grip strength and ability to perform muscular activities, should be an important issue for antenatal care providers in order to secure good and safe childbirth and care for the newborn.

Our former study, performed in springtime, showed a very poor grip- and leg strength and severe vitamin D deficiency (<25 nmol/L) in 90% of pregnant or newly pregnant Somali women living in Sweden. Similar findings were found in 10% of the native pregnant women. Grip strength was positively associated with 25-OHD levels also when adjusted for country of birth, age and height [1].

Muscles respond to mechanical loading and metabolic distress [2–4], and vitamin D plays a crucial role in muscle function [5], calcium absorption and in maintaining homeostasis [6]. To note, impaired muscle function may be present before the biochemical signs of bone disease appears [7, 8]. Few or no studies have examined adherence to supplemental vitamin D medication in long-term and its effect on muscle strength in pregnant women focusing on those wearing veiled clothing [9].

The study aims here were to explore adherence to prescribed supplemental vitamin D in pregnant and recently pregnant women with vitamin D deficiency and its effects on grip strength and upper leg performance in Somali (target group; TG) and Swedish women (reference group; RG) from spring through winter.

## Methods

### Design

A pre- and post-treatment study design was used. The study was initiated in late spring (baseline) to take advantage of the low UV B radiation conditions during winter. We chose a retrospective inclusion design that enabled us to complete blood sampling and physical tests within a limited period of time. Thus, the baseline sample included women in all trimesters as well as recent mothers. A 4-month check-up was performed during autumn (Time I). The primary outcome data were collected at the 10-month follow-up, after winter (Time II).

### Setting

The study took place in a primary care antenatal clinic in central Sweden (latitude, 60th parallel north).

### Recruitment

Study participants were recruited from women registered at the antenatal clinic during 2010 [1]. We excluded women <18 years of age, women born in countries other than Somalia and Sweden and women with severe disorders such as severe metabolic, medical or mental disease (ex. severe diabetes or psychosis). Information material was translated into Somali and read aloud as needed. Veiled clothing was defined as clothing covering the woman’s arms, legs and head. A total of 217 women were randomly selected based on primi- or multiparity and recruited via letter and telephone. In total, 140 women agreed to participate in the study and had initial blood tests. One week later, 123 women attended the baseline physician consultation for physical performance tests, supplemental vitamin D prescriptions when needed and brief lifestyle information.

### Study sample

Of the 123 women who participated at baseline, 89 had 25-OHD ≤50 nmol/L. Included in analyses are 46 Somali women in the target group (TG) and 25 Swedish women in the reference group (RG) who also attended the 10-month follow-up measurements (Time II). Liver enzyme levels were normal at baseline.

### Intervention

A vitamin D3 supplement containing calcium (800 IU = 20 μg vitamin D3 and 500 mg calcium carbonate) was prescribed. The over-the-counter price was 309 SEK per 180 tablets (approximately 44 USD) and the women paid these themselves. Two tablets daily was prescribed for the women with 25-OHD <25 nmol/L and once daily for those with 25–50 nmol/L of 25-OHD. These doses followed the recommendations for pregnant women [10].

### Data collection

Professional interpreters supported data collection from Somali women. Anthropometric measurements were collected. Data on breastfeeding and gestational age were noted and Body Mass Index (BMI) was calculated. Physical activity was self-reported on a 3-point scale: seldom, sometimes or often (≥3 times weekly). Questions about medication and side effects were asked during the follow-up meetings. The research physicians were blinded to the blood test and previous test results during the consultations.

### Blood samples

Venous blood samples were drawn and centrifuged. Serum vitamin D (25-OHD) was measured using the Liaison 25 OH Vitamin D total assay (DiaSorin, Stillwater, USA) at Akademiska Laboratoriet, Uppsala, Sweden. The other assays (s-ALAT, s-ALP, fP-PTH and serum albumin corrected Ca) were measured using Abbott Architect ci8200 (Abbott Laboratories, Illinois, USA) at the Department of Clinical Chemistry, Falun Hospital, Sweden. Alkaline phosphates (s-ALP), fasting parathyroid hormone (fP-PTH) and free serum calcium were analysed to monitor metabolic skeletal activity. The same laboratories and assays were used on all test occasions. Serum 25-OHD was used as the indicator of vitamin D status and fP-PTH and s-ALP were indicators of bone turnover at baseline and 4 months.

Haemoglobin and glucose were measured in the baseline blood samples to screen for non-vitamin-D-related reasons for fatigue and muscular weakness.

### Grip strength and upper leg performance

Physical performance tests in the hands and upper legs were chosen because they reflect everyday uses. Test outcomes were rated by the same researcher physicians on all occasions.

### Grip strength

Maximal grip strength held for 10 s (Newton, N) for testing endurance was measured three times in each hand using a hand dynamometer at each assessment (GRIPPIT AB Detektor, Gothenburg, Sweden) [11]. The mean value was used in the analyses (M-max grip). Also peak grip strength was measured at all times.

### Upper leg performance

Four upper leg tests were rated by the research physicians on a 3-point ordinal scale: able (0), with help (1) or unable (2), and later categorized as: able (0) or unable (1 and 2). The tests included:

- Squatting. The person squatted and rose once.
- Standing on one leg for 30 s.
- Hip lifting (Trendelenburg’s test). The person held one hand high up on a wall and lifted the opposite leg for 30 s.
- Rising from a chair five times in a row while folding arms across chest. This test was later omitted from analyses because all participants accomplished it.

### Medication

Daily consumption of prescribed tablets and compliance to the medication dosage were asked for at follow-up appointments and total number of tablets consumed for 10 months was estimated based on this information.

### Ethical approval

Written consent was given by participants. The study was approved by the regional ethics committee in Uppsala, Sweden (D 2010/140 and 2010/140/1).

### Power calculation

Statistical power was calculated based on an upward clinically relevant mean difference in grip power of 50 N (Std 40). On this basis, at least seven participants were needed in the TG and 17 in the RG (mean change 40, Std 50) for a two-sided *t* test to reach 90% power with an alpha error of 5%.

### Statistical analyses

The study sample was divided into two groups, TG and RG. Levels of 25-OHD were examined and categorized according to standards as: un-measurable <10 nmol/L (coded as 9 in statistical calculations), 10–24 nmol/L or 25–50 nmol/L. Mean values (intervals) and standard deviations (Std) were calculated. We used Chi square statistics for nominal data and Mann–Whitney *U* test to test for significant differences in median and mean values between groups for both non-normal and normally distributed variables. Non-parametric tests for significant differences between pre–post values were conducted using McNemar’s test (for dichotomous data) or Wilcoxon signed-ranks test (for ordinal or interval data). Uni-variate associations were explored using Spearman rho to assess correlations between the number of tablets taken and the difference scores between Baseline and Time II on 25-OHD, M-max grip and upper leg performance.

We used multiple linear regression with stepwise exclusion to estimate the independent variables that was best associated with the change of grip strength from baseline to Time II.

We used binary regression to calculate odds ratios (OR) with 95% confidence intervals (95% CI) as predicting positive changes in upper leg physical performance in the TG.

Independent variables age, total tablets taken, height, increased physical activity and 25-OHD increase were categorized as below and above their mean values.

Data were analysed using SPSS, version 20.

## Results

Despite reminders, 5 of the 51 (10%) in the TG and 15 of 40 (38%) in the RG did not participate further (drop-outs). They did not differ significantly in any aspects from those who continued. The five TG women had not given any motives but some of the RG women said they felt fine and therefore did not want to participate further.

In addition, the gelatine component of the tablets had worried a few TG women but they were satisfied when told that it was from a bovine source. A few women reported initial gastric irritation.

Table 1 shows that the 25 RG women were older (*p* < 0.05), taller (mean height 167 cm vs. 161 cm, *p* < 0.001), more educated (mean 14 years vs. 3 years, *p* < 0.001) and had lower parity (1 vs. 3, p < 0.001) than the TG women. All, except two TG women, had very low or un-measurable (37%) 25-OHD levels with an estimated mean of 16 (median 12 nmol, not in table), compared with 39 nmol/L in the RG, and with fP-PTH and s-ALP levels above the reference limits.

**Table 1:** Baseline characteristics for entire sample and participants in the target and reference groups. Values are means with standard deviation (Std), number (n) and frequencies (%)

	Target group		Reference group	
	All	Participants	All	Participants
Number, *n* (%)	51	46 (90.2)	40	25 (62.5)
Age, years	28.3 (6.4)	28.0 (6.5)	30.4 (5.4)	30.6 (5.7)
Parity, *n* (%)	3.4 (2.7)	3.1 (2.5)	1.3 (1.1)	1.4 (1.1)
Education, years	3.4 (3.4)	3.5 (3.5)	13.9 (2.5)	14.3 (2.2)
Height, cm	161.5 (5.2)	161.4 (5.1)	167.0 (6.3)	167.2 (6.1)
25-OHD nmol/L	15.4 (7.8)^a^	15.8 (9.7)^a^	37.8 (9.5)	38.8 (10.3)
s-Ca, mmol/L	2.27 (0.12)	2.27 (0.12)	2.31 (0.07)	2.31 (0.06)
fP-PTH pmol/L	12.7 (11.0)	13.0 (11.5)	4.71 (2.49)	4.87 (2.90)
s-ALP, ukat/L	2.00 (1.40)	2.02 (1.45)	1.58 (0.77)	1.56 (0.76)

Reference ranges: fP-PTH, 1.1–6.9 pmol/L; s-albumin corrected calcium, 2.15–2.50 mmol/L; s-ALP, 0.60–1.80 ukat/L

^a^25-OHD values <10 nmol/L (unmeasurable) replaced with 9 for 17 women (34%) in the entire sample and *n* = 17 (37%) among target group participants

Note: NS. drop-outs (*n* = 5 TG; *n* = 15 RG) vs. participants (Mann–Whitney *U* test)

Seventeen (37.0%) women in TG were pregnant (mean months 6.2, Std 1.3) compared to 15 (60.0%) in RG (mean months 6.0, Std 1.7). In TG 28 (60.9%) women were breastfeeding compared to 9 (36.0%) in RG.

### Check-up Time I at 4 months (data not shown in tables)

Objectively, the 38 TG women had significantly improved their *peak* grip strength from a mean of 199 N (Std 59) to 214 N (Std 49) (*p* = 0.037, Wilcoxon signed-ranks test) and were more able to squat effortlessly (*p* < 0.001). At this time point, no TG woman had an immeasurable 25-OHD level; their group mean was 47 (Std 28) nmol/L vs. the initial estimated mean of 15.8 (Std 9.7) nmol/L (*p* < 0.001). Furthermore, their mean fP-PTH levels decreased to 8.3 (Std 3.1) from 13.0 (11.5) pmol/L at baseline (*p* < 0.001). The RG women also had increased 25-OHD to 64 nmol/L (Std 26) vs. 39 (Std 10) at baseline (*p* < 0.001).

### Check-up Time II at 10 months

Eight (17%) in the TG vs. two (8%) of the RG reported having not taken any supplement. Thus, there was a large variation in the calculated mean total intake of tablets at Time II : *n* = 322 (Std 239) in the TG and *n* = 211 (Std 136) in the RG.

Mean 25-OHD levels were stable compared with Time I (47 to 49 nmol/L TG; 64 to 67 nmol/L RG).

The increased levels of 25-OHD correlated with total tablet intake in the TG (rho. 0.53, p < 0.001).

Many women reported increased physical activity compared to baseline; 21/46 in TG (ns) and by 14/25 in RG (p < 0.001).

Table 2 shows baseline and Time II levels of 25-OHD, grip strength and the number of women unable to perform the upper leg performance tests at baseline and Time II. At time II, the TG women had significantly higher 25-OHD and grip strength and the number of women unable to keep their hip up and to squat decreased significantly. More than half (26 of 46; 57%) of the TG women improved their squatting ability. None improved on the one-leg standing task. Likewise, the RG women had significantly higher 25-OHD values at Time II, as well as a stronger grip strength (mean 257 N vs. 297 N, *p* = 0.003). This concerned even the women in the higher strata.

**Table 2:** Mean (Std) values for the target and reference groups at baseline (time 0) and after 10-months (time II) of 25-OHD nmol/l and grip strength; number and frequencies of those unable to perform the upper leg tests

	Target group			Reference group		
	*N* = 46			*N* = 25		
	Time 0	Time II	*p*	Time 0	Time II	*p*
25-OHD mean (Std)	15.8 (9.7)^a^	49.2 (35.4)	.001	38.8 (10.3)	67.5 (26.7)	.001
Grip strength, N (mean, Std)	153 (55)	188 (57)	.001	257 (68)	297 (58)	.003
Unable to squat, *n* (%)	35 (76.1)	9 (19.6)	<.001	0 (0)	1 (4.0%)	1.000
Unable to stand on one leg, *n* (%)	11 (23.9)	11 (23.9)	1.000	1 (4.0)	0 (0)	1.000
Unable to keep hip up, *n* (%)	10 (21.7)	1 (2.2)	.004	1 (4.0)	0 (0)	1.000

^a^ Mean 25-OHD estimated at 9 for *n* = 17 women with unmeasurable 25-OHD (<10 nmol/L) at Time 0. Significant differences before and after were calculated by Wilcoxon signed-ranks test for interval data and McNemar’s test for dichotomous data

Table 3 shows before and after values for strength and 25-OHD in 25-OHD categories of compliers (adhered to medical advice) and non-compliers after 10 months of treatment. In 25-OHD category 10–24 nmol/L, TG women who took medication (compliers) increased their grip strength with 28% (162 N–208 N, *p* < 0.001) and 25% (118 N–148 N, *p* = 0.039) in category <10 nmol/L. Two thirds (64%) for the groups of TG women with <10 or 10–24 nmol/L who adhered to medical advice (compliers) increased their leg strength significantly.

**Table 3:** Before and after values of 25-OHD and physical tests at baseline and 10 months (Time II) by target and reference groups (participants) and categories of 25-OHD concentrations (nmol/L) groups based on 25-OHD levels at baseline and compliers or non-compliers of supplement intake. Statistical significans with Wilcoxon

	Target group					Reference group	
N	46					25	
	Compliers			Non-compliers		Compliers	
n (%)	38 (82.6%)			8 (17.4%)		23 (92%)	
25(OH)D category^a^	<10	10–24	25–50	<10	10–24	10–24	25–50
n	13	21	4	4	4	5	18
25(OH)D, mean (Std)							
Baseline	9.0 (0.0)	16.1 (5.0)	39.0 (15.1)	9.0 (0.0)	19.8 (3.1)	22.5 (1.6)	42.0 (6.7)
10 m	50.5 (36.1)	57.9 (39.0)	60.3 (8.0)	15.0 (4.1)	22.6 (10.9)	66.3 (29.0)	66.6 (28.2)
p	**0.001**	**<0.001**	0.068	0.102	0.715	**0.043**	**0.001**
*Physical performance*							
Grip (N), mean (Std)							
Baseline	118 (64)	162 (36)	193 (103)	166 (37)	171 (23)	242 (51)	262 (76)
10 m	148 (65)	208 (46)	219 (54)	168 (37)	201 (43)	267 (34)	310 (59)
p	**0.039**	**<0.001**	0.465	0.593	0.144	0.080	**0.010**
Inability to: Squat, n(%)							
Baseline	11 (84.6)	13 (61.9)	3 (75.0)	4 (100)	4 (100)	0 (0.0)	0 (0.0)
10 m	0 (0.0)	4 (19.0)	1 (25.0)	2 (50.0)	2 (50.0)	1 (20.0)	0 (0.0)
p	**0.001**	**0.012**	0.500	0.500	0.500	1.000	
Stand on one leg, n (%)							
Baseline	3 (23.1)	5 (23.8)	0 (0.0)	2 (66.7)	1 (25.0)	0 (0.0)	1 (5.6)
10 m	3 (23.1)	3 (14.3)	2 (50.0)	2 (66.7)	0 (0.0)	0 (0.0)	0 (0.0)
p	1.000	0.688	0.500	1.000	1.000		1.000
Keep hip up, n (%)							
Base line	4 (30.8)	4 (19.0)	0 (0.0)	1 (25.0)	1 (25.0)	0 (0.0)	1 (5.6)
10 m	0 (0.0)	0 (0.0)	0 (0.0)	1 (25.0)	0 (0.0)	0 (0.0)	0 (0.0)
p	0.125	0.125		1.000	1.000		1.000

Note: ^a^Categorized 25-OHD values due to unmeasurable values and treatment strategies at baseline

Bold data are significant

**Fig. 1:**
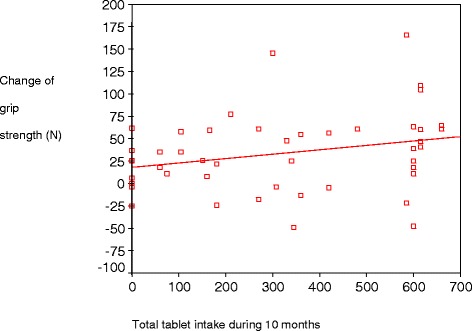
Scatter plot for total tablet intake and change in grip strength (M-max grip, measured in Newton [N]) from baseline to Time II (10-month follow-up) for the target group (TG) women, *n* = 46. Spearman’s rho 0.32, *p* = 0.032. M-max grip was mean maximal grip held for 10 s

Figure 1 illustrates a scatterplot displaying the association between total tablet intake and M-max grip increase (rho 0.317, *p* = 0.032).

Table 4 shows the final effect model for grip strength change. Total tablet intake was positively associated with improved grip strength (*p* = 0.027), adjusted for height (*p* = 0.036).

**Table 4:** Final model using multiple linear regression for improved Mean maximal grip strength held for 10 s at 10-month follow-up (time II). Unstandardized B with 95% confidence intervals (95% CI) (*N* = 71)

	B	*p*-value	95% CI for B	
			Lower	Upper
Total tablet intake	.067	0.027	.008	.127
Change in 25-OHD	-.338	0.092	–.733	.056
Height	.060	0.036	.157	3.966

Adjusted R^2^ 0.100. Variables entered in the first model: age, height, country of birth, total tablet intake, 25(OH)D change and physical activity change

Table 5 shows the OR for improved squatting ability in the target group with 95% CI in a final model. The OR for intake of one tablet at least once daily for 10 months (300 tablets or more) showed an OR of 16.0 (95%CI 1.8–144.6).

**Table 5:** Odds ratios with 95% confidence intervals (95% CI) for improving squatting ability in target group. Final full model

	*p*	OR	95% CI	
			Lower	Upper
Total tablet intake > 300	.014	16.0	1.8	144.6

Variables entered at step one: age > 28 years, height > 161 cm, decreased BMI, increased 25-OHD > 33 nmol/L, tablet intake > 300, increased physical activity

Nagelkerke R square: Step 1; 0.359 Final step; 0.315

## Discussion

To sum up, the adherence to the prescribed supplement regiment was good in this target group of women, with critically low 25-OHD levels and very poor muscular performance at baseline. Taken all participants together, the improved grip strength was significantly associated to the number of tablets taken and this was also the case with improved upper leg performance in the target group.

### What is new?

To our knowledge, there are no previously published real life studies of the usefulness of prescribing supplemental vitamin D for improving grip strength and upper leg performance to low-educated new immigrant and veiled non-Western pregnant (and recently pregnant) women with severe vitamin D deficiency. In addition, there are few reports of supplement efficacy to younger women with less severe vitamin D deficiency to improve their grip strength.

### Discussion of results

This was a real life study in a multicultural primary care made in cooperation with the antenatal care. We targeted women at special risk for complications due to severe vitamin D deficiency. Path ological bone turn-over at baseline was significantly reduced already after a few months.

Like most Western countries, Sweden as a country has an influx of refugees from the global conflict regions thus changing the ill-health patterns in the population and brings up a need for new clinical practices [12, 13]. Non-adherence to medication and advice can be due to lack of cultural competence from a care-giver perspective leading to that clinical needs are missed [14] such as the deficient levels of vitamin D (25-OHD) in pregnant non-Western women living in Europe [15–17], in adolescent girls in England [18], and in post-partum mothers [15]. Lack of vitamin D also occurs in high frequency in native populations of women in Beijing [19] and Denmark [20] while Swedish women in general practice seldom have levels below 25 nmol/L [21]. Notably, no clinical data were provided in any of the above mentioned studies.

A standard estimate of appropriate serum 25-OHD is 50 nmol/L [6, 8, 22, 23]. Our TG women had much lower values and even immeasurable 25-OHD (i.e., <10 nmol/L) levels and also very weak hands and legs. With supplementation, their 25-OHD levels increased to 50 nmol/L 25-OHD which apparently had contributed to improve their grip strength 25% on the average. Yet, their mean grip strength value was only 188 N, which still was 80 N below the “normal” mean value for healthy Swedish women (268 N for 20–29 years) [11]. Also, a quarter of the TG women could not stand on one leg at any time. This finding is unclear and worthy of further investigation, as are the other possible explanations for weak handiness. One fifth of the TG women had a positive Trendelenburg’s test at baseline, compared with only a few at Time II not possible to relate directly to tablet intake probably due to small number of persons.

Long recognised as important for bone health, vitamin D has attracted interest also for its possible non-skeletal benefits. Whether supplemental vitamin D lowers and at what dose is required to reduce health hazards is uncertain [24]. More is not necessary better, as shown here when a moderate dose of 800 or 1600 IU vitamin D3 was beneficial also for women with very poor status. In a multicultural clinical trial like this it is impossible to correct for all confounders. Here we previously have accounted for many likely confounders but only physical activity was found relevant.

Grip strength is a measure of upper extremity function commonly used as a general indicator of frailty among older adults. Here, improved grip strength and ability to squat correlated to the number of tablets consumed rather than the laboratory levels of 25-OHD, and so likely indicating the importance of calcium content when it comes to improving skeletal muscle function.

The cut-off points for S-25-OHD were from the laboratory [10]. Our laboratory consistently returned values 10–20% lower than the precise LC-MS reference methods used at other laboratories [25].

Some authors have suggested 75 nmol/L of 25-OHD as optimal for soft tissue health [10, 22, 26, 27]. Our RG women with better baseline values (25–50 nmol/L 25-OHD) also increased their 25-OHD levels significantly and increased their grip strength from subnormal levels (mean 257 N) to the normal mean value for 30–39-year-old Swedish women [11].

Clinically, both appearance and gait among the TG women had improved by Time I. Here, we assessed physical performance as separate tests and not a summary score that sometimes is used to illustrate a broad measure of physical performance [27]. We considered separate measures of performance easier to manage in a multicultural clinical practice.

Gestational age and breastfeeding indicating also oestrogen status may theoretically influence performance of physical tests but no such relation was found here, nor in our previous study [1].

The association between serum vitamin D, supplemental vitamin D and physical performance is still poorly understood. Our low correlation values between 25-OHD and improved grip strength could be partly explained by the conservative replacing method for immeasurable 25-OHD values and/or a total body calcium deficiency. Participants also reported an increase in their physical activity but this could not be used to explain their improved test results.

### Strengths and limitations

This study is by us considered to have a good ecological validity. A problem we faced was the difficulty of enrolling pregnant and recent mothers. Language and cultural differences were additional barriers, but in spite of this most of the TG women enrolled when the Somali assistant nurses contacted them.

To note, the dropout rate was higher among the more affluent reference women than among the marginalized target group women. This may have distorted some of the results, which must in any case be interpreted with caution due to the small sample size from a single site.

Our results cannot be generalized but should be possible to transfer to similar patient settings as here, and they might also be relevant for individuals with low UV exposure for prolonged periods.

Regrettably, a randomized controlled intervention study was not possible to perform due to ethical concerns. Instead, we performed this *in situ* study in a single setting with a limited number of participants at risk.

### Comparison with other studies

Hypovitaminosis D can jeopardize development of the foetus and cause neonate hypocalcaemia, which motivated us to focus on pregnant women in our study [10]. Many studies conclude that vitamin D deficiency is common in middle Eastern female immigrants but that their response to prescribed vitamin D dosages is often inadequate [28].

This real life study included persons with minimal UV-exposure during an extended time. Here, the estimated level increase of 25-OHD was 31 nmol/L per 800 IU (20 ug) vitamin D3 plus 500 mg calcium in the TG and even 41 nmol/L in RG compared to a lower increase of 25 nmol/L at intake of 1000 IU according to previous literature [29]. The comparatively higher result in RG could be due to more UV-exposure but this needs further investigation. Our results may provide a formula for calculating the amount of supplemental intake in pregnancy during long-term to increase 25-OHD levels to normal in similar groups of persons with low UV-exposure.

Cutaneous synthesis of vitamin D3 decreases with age inducing interest in the vitamin D status and lower extremity functioning of older adults [8] but the relationship between muscle strength and supplemental vitamin D has been discrepant with regard to muscle strength and function [30].

Cross-sectional studies have shown a positive relationship between muscle strength, performance and 25-OHD levels among postmenopausal women having levels below 60 nmol/L and in hip-fracture patients [31, 32]. In women 19–29 years with <50 nmol/L 25-OHD, more physical activity was associated with both good grip strength and higher serum 25-OHD [22].

Intervention studies have provided diverging results depending on the study population and intervention methods. On the positive side are studies of older adults [8] and athletes with sub-optimal 25-OHD [22] in whom supplemental vitamin D with calcium improved muscle strength and athletic performance. An important point here is that a combination of calcium and vitamin D was found to be superior for improving muscle function compared with calcium alone [33]. In a young adult sample, Danish investigators found improved quadriceps muscle power after vitamin D treatment in veiled Arabic adult women [7]. On contrary, a Norwegian study of non-Western adults, many of whom were from Somalia, found that men and women with on average 25-OHD of 27 nmol/L did not benefit from 4 months of supplemental vitamin D, notably without calcium, as measured by their power to jump, grip, or rise from a chair [34]. Furthermore, a study of young women found no relationship between increased 25-OHD and improved muscle strength after having received vitamin D and calcium [35]. However, small beneficial effects of supplemental vitamin D on balance, gait or sit-to-stand performance were found in community-dwelling older women [36].

Adherence studies of supplemental vitamin D intake are few.

### Clinical significance

In clinical practice, signs such as a weak handgrip, the inability to squat or a waddling gait (also called Trendelenburg gait) indicate a severe vitamin D deficiency in high-risk groups.

Giving supplements containing vitamin D and calcium may help improve function and seem to be well accepted, but support from native-speaking assistants might be needed to improve adherence. Repeated measurements of 25-OHD appear to be of little use and might be replaced by tablet intake estimation and simple physical performance tests.

### Future studies

Our findings warrant further investigation, including studies focusing on methods to improve adherence to supplemental prescriptions where also costs of supplements, attitudes and behaviour-related socio-cultural factors should be explored by e.g., using qualitative research methods. Future studies could also focus on delivery outcome, efficacy in daily infant care, balance and osteomalacia.

## Conclusions

Adherence to supplemental vitamin D and calcium should be encouraged as an even moderate intake was associated to improved grip strength and upper leg performance, which was particularly useful for the women with critically 25-OHD deficiency and poor physical performance at baseline.
